# Sex Differences in the Relationship Between Nucleus Accumbens Volume and Youth Tobacco or Marijuana Use Following Stressful Life Events

**DOI:** 10.29245/2578-2959/2024/2.1305

**Published:** 2024-03-18

**Authors:** Shervin Assari, Payam Sheikhattari

**Affiliations:** 1 Department of Internal Medicine, Charles R. Drew University of Medicine and Science, Los Angeles, CA, USA; 2 Department of Family Medicine, Charles R. Drew University of Medicine and Science, Los Angeles, CA, USA; 3 Department of Urban Public Health, Charles R. Drew University of Medicine and Science, Los Angeles, CA, USA; 4 Morgan State University, Baltimore, USA

**Keywords:** Sex differences, Youth, Adolescence, Stress, Substance use

## Abstract

**Background:**

Exposure to stressful life events (SLEs) can upset balance and affect the healthy brain development of children and youths. These events may influence substance use by altering brain reward systems, especially the nucleus accumbens (NAc), which plays a key role in motivated behaviors and reward processing. The interaction between sensitization to SLEs, depression, and substance use might vary between male and female youths, potentially due to differences in how each sex responds to SLEs.

**Aims:**

This study aims to examine the effect of sex on the relationship between SLEs, Nucleus Accumbens activity, and substance use in a nationwide sample of young individuals.

**Methods:**

We utilized data from the Adolescent Brain Cognitive Development study (ABCD), a longitudinal study of pre-adolescents aged 9–10 years, comprising 11,795 participants tracked over 36 months. Structured interviews measuring SLEs were conducted using the Kiddie Schedule for Affective Disorders and Schizophrenia (K-SADS). Initial linear regression analyses explored if SLEs could predict volumes of the right and left NAc. Subsequently, Cox regression models were used to investigate how SLEs and NAc volume might predict the initiation of tobacco and marijuana use, with the analysis stratified by sex to address potential sex differences.

**Results:**

Our findings reveal that SLEs significantly predicted marijuana use in males but not in females, and tobacco use was influenced by SLEs in both sexes. A higher number of SLEs was linked with decreased left NAc volume in males, a trend not seen in females. The right NAc volume did not predict substance use in either sex. However, volumes of both the right and left NAc were significant predictors of future tobacco use, with varying relationships across sexes. In females, an inverse relationship was observed between both NAc volumes and the risk of tobacco use. In contrast, a positive correlation existed between the left NAc volume and tobacco and marijuana use in males, with no such relationship for females.

**Conclusion:**

This study underscores that the associations between SLEs, NAc volume, and subsequent substance use are influenced by a nuanced interplay of sex, brain hemisphere, and substance type.

## Introduction

Stressful life events (SLEs) during youth development are described by a wide range of terminologies such as Adverse childhood experiences (ACE)^1,2^, Early Childhood Adversity (ECA)^3^, Early Life Adversity (ELA)^4^, and Early Life Stress (ELS)^5,6^. Regardless of the term used, all of these describe common exposures of children to life events that require effortful coping^7,8^.

Two out of each ten children face SLEs such as emotional abuse, sexual abuse; or physical abuse during their developmental phase^4,9^. Exposure to SLEs increases the risk of mental and physical health problems such as substance use^9–11^. SLEs tend to be comorbid with economic challenges, poor parenting^12^ and high risk parents^13^. In their 1998 landmark study, Felitti and colleagues established a strong predictive role of exposure to SLEs and worse adult health^14^.

Given that youth is a stage of vulnerability to SLEs^15,16^, SLEs are common, and SLEs may have lasting consequences on healthy brain development^17,18^ many scholars have turned to better understand the nuances about the relationship between SLE and substance use^2,19^ through the role of brain networks and systems such as reward regulation.

According to the stress sensitization model^20,21^, SLEs may sensitize individuals to heightened sensitivity and reactivity to subsequent stress^22^. This model proposes an increased sensitivity to SLEs in females who are regularly exposed to more SLEs. According to stress habituation model^23^, low-level but chronic exposure to SLEs reduces vulnerability to future stress, as the individual mobilizes the assets and resources that can be used to buffer the stress^24^. According to this hypothesis, females who are more exposed to SLEs will become more ready to handle the next stressors.

However, no one size fits all and factors such as sex may alter some of the interconnection between SLE, brain (reward system), and substance use^25–27^. From one side, SLEs are more common in females than males^28,29^. From another side, substance use is more common in males than females. Finally, sensitivity to SLEs may also differ between males and females. In addition, some evidence suggests that sex may alter the brain processes that are involved in the regulation of reward and motivated behaviors.

Although some reviews have included an examination of sex differences in the link between SLE and psychopathology^11,18,30,31^, no review has explored the biology leading to potential brain pathways and structures that may mediate the relationships among these variables.

Recently, we developed a model to study sex differences in the effects of SLEs on substance use via brain mechanisms including but not limited to the reward system^32^. We provided a historical review of studies that have tested sex differences in the effects of SLE on brain and substance use^32^. The model suggested that depending on sex, sex hormones may increase (estrogen) or decrease (testosterone) cortisol levels and modulate response to stress signals in emotion regulation and brain reward centers. As such, youth who experience increased mental stress may use substances in order to cope with SLEs, however, in a sex-specific manner. This model proposes mental health as the mediating factor and sex as the moderating factors for the effects of SLEs on the substance use of youth^32^.

Nucleus accumbens (NAc) is a core element of the reward system of the brain and is located in the basal ganglia^33–38^. NAc has implications in reward seeking, response to cues, motivated behaviors, and reinforcement^39–44^. As such, any changes that occur in the structure, microstructure, or function of the NAc is expected to predict altered reward seeking behaviors^45–49^. Thus, NAc change is correlated with the pathogenesis of addiction to food^33,50–52,53^ and drugs^54–57^.

Alterations in the nucleus accumbens (NAc) may underlie a broad spectrum of addictive disorders characterized by heightened cue sensitivity and a greater tendency for reward seeking^41,50,58–60^. Investigations in both animal^61^ and humans^62–67^ have revealed that both the functionality and size of the NAc undergo modifications in conditions of heightened reward sensitivity and the presence of addiction^53,62,68–75^. As a critical brain region implicated in the motivation for incentive-seeking, changes in the NAc are consistently observed across addiction and substance use scenarios^61^. Specifically, cues associated with food and drugs trigger a pronounced dopamine response in anticipation of rewards^53^, leading to an increased drive towards seeking these rewards^49,65,76–78^. Despite our existing knowledge on the NAc’s involvement in addiction development, further research is imperative due to several limitations: predominant reliance on animal studies, the small sample sizes of human studies, the artificial nature of highly controlled experimental settings, and the prevalent use of cross-sectional study designs^62–67^. There’s a marked need to explore the predictive value of baseline NAc volume regarding the onset of substance use among youths in extensive, nationally representative studies involving diverse populations.

Given the NAc’s role in the brain’s dopaminergic reward systems^79^, identifying changes within the NAc could aid in distinguishing variations in food and drug seeking and preference behaviors^80,81–87^. Furthermore, research has established a correlation between NAc modifications and shifts in motivated behaviors, encompassing the regulation and pursuit of cues, food, drugs, addiction, and obesity^81–85^. Insights from research both external^81–85^ and internal to the Adolescent Brain Cognitive Development (ABCD) study suggest the potential of NAc alterations as predictive markers for reward-related disorders in the future^88–94^.

### Aims

Our research aimed to explore how gender influences the relationship between SLEs, the NAc, and substance use among a nationwide cohort of young individuals. We posited that experiencing a high number of SLEs would correlate with reduced NAc volume, potentially leading to increased substance use in the future. Specifically, we anticipated that this effect would be more pronounced in males, in line with findings from previous research^32,95^.

## Methods

### Study Design and Context

This study is a secondary analysis based on data from the Adolescent Brain Cognitive Development (ABCD) study^96–100^, a comprehensive investigation into the neurocognitive processes associated with the onset of substance use during the transition from childhood to early adulthood. The ABCD study is longitudinal, tracking substance use biannually and conducting major assessments every two years. Further information about the ABCD study’s methodology is detailed in other publications^96,101^.

### Participants and Method of Selection

The ABCD study has monitored over eleven thousand individuals aged 9 to 10 years at the start of the study, recruiting participants from 21 sites across 19 cities in 15 U.S. states. While not utilizing a random sampling method, the ABCD cohort closely mirrors the demographic composition of U.S. children aged 9–10 during the years 2016 to 2018, with schools serving as the primary recruitment venues^102^.

### Sample for Analysis

The subset of the cohort included in our analysis comprised 11,795 pre-adolescents who had follow-up data on subsequent substance use. Eligibility for this analysis required participants to be aged 9 to 10 years at baseline, have follow-up substance use data, and possess valid demographic information.

### Neuroimaging Approach

We analyzed pre-processed neuroimaging data from the ABCD study^96–100^, utilizing both functional and structural MRI scans, including resting-state and task-based fMRI. Our analysis focused on resting-state fMRI, calculating the beta correlation between the frontoparietal network and the accumbens-area region of interest (ROI). Imaging data were collected using 3 Tesla (T) scanners from Siemens Prisma, General Electric 750, and Phillips, all equipped for multiband echo-planar imaging (EPI) acquisitions. The procedure included initial localizer scans followed by T1-weighted structural and T2-weighted functional MRI acquisitions. The ABCD study’s comprehensive imaging approach yielded a wide array of data from adolescents across the U.S., with all structural and functional MRI data being pre-processed and ROI data sourced from the NIMH Data Archive (NDA). We specifically examined resting-state fMRI data from the ABCD study, with additional imaging protocol details available in the referenced documentation^103–105^.

### Variables of the Study

#### Outcomes

##### Tobacco and Marijuana Use.

We assessed tobacco and marijuana use every six months^99^. Although questions about the context of first use were asked at one point, they were not included in our study. At baseline (Y0), participants reported their lifetime substance use via an online Timeline Follow-Back (TLFB)^106^ interview for the past six months (at baseline) or since the last assessment (for subsequent evaluations). Our analysis encompassed a range of substances, with semi-annual telephone follow-ups enriching the data on yearly substance use. For our purposes, substance use was categorized into experimental (e.g., minimal tobacco or marijuana use) and initiation stages (defined as >1 instance of use). We developed three variables to track the onset of tobacco and marijuana use identified six months or more after the study’s commencement.

#### Independent Variables

##### SLEs.

Parents were interviewed regarding the trauma experienced by the child. The Kiddie Schedule for Affective Disorders and Schizophrenia (K-SADS)^107^ was used to measure trauma. This is a semi-structured interview aimed at the early detection of high-risk youth. The items included: (1) “A car accident in which your child or another person in the car was hurt bad enough to require medical attention”, (2) “Another significant accident for which your child needed specialized and intensive medical treatment”, (3) “Witnessed or caught in a fire that caused significant property damage or personal injury”, (4) “Witnessed or caught in a natural disaster that caused significant property damage or personal injury”, (5) “Witnessed or present during an act of terrorism (e.g., Boston marathon bombing)”, (6) “Witnessed death or mass destruction in a war zone”, (7) “Witnessed someone shot or stabbed in the community”, (8) “Shot, stabbed, or beaten brutally by a non-family member”, (9) “Shot, stabbed, or beaten brutally by a grown up in the home”, (10) “Beaten to the point of having bruises by a grown up in the home”, (11) “A non-family member threatened to kill your child”, (12) “A family member threatened to kill your child”, (13) “Witness the grownups in the home push, shove or hit one another”, (14) “A grown up in the home touched your child in his or her privates, had your child touch their privates, or did other sexual things to your child”, (15) “An adult outside your family touched your child in his or her privates, had your child touch their privates or did other sexual things to your child”, (16) “A peer forced your child to do something sexually”, and (17) “Learned about the sudden unexpected death of a loved one”. Response items for each item were 0 (no) or 1 (yes). We counted the number of traumatic events, and, given the extreme skewness of the count of traumatic events, we calculated a variable as zero traumatic events, one traumatic event, and two or more traumatic events (Cronbach’s alpha = 0.637)^108^.

##### Right and Left NAc Volume.

By employing structural magnetic resonance imaging (fMRI), we used the volume of ASEG region of interest (ROI) corresponding to the right and left accumbens-area. These measures quantify right and left NAc volumes at baseline.

##### Age.

Age (months), calculated as the difference between birth and the time of enrollment to the study, measured in months, was reported by parents.

##### Sex.

A dichotomous variable, sex was coded as follows: males = 1, females = 0.

##### Race/Ethnicity.

Race/ethnicity, a self-identified variable, was a categorical variable: non-Latino White = 1, any other racial/ethnic group = 0 (reference category) that included Blacks, Latino/Hispanic, Asian, and mixed/other races.

##### Parent Education (yrs).

Parents reported their years of schooling. This variable was operationalized as a continuous (interval) variable ranging from 0 for no formal education to 21 for doctoral degrees^109^.

##### Family Composition.

Parents reported the number of parents in the household. This variable was operationalized as a categorical variable with 0 for one and 1 for two parent households.

##### Family Income.

Family income was a 1–10 interval measure where a higher score indicated higher income. The total combined family income in the past 12 months was asked. Responses were 1 = less than $5000; 2 = $5000+; 3 = $12,000+; 4 = $16,000+; 5 = $25,000+; 6 = $35,000+; 7 = $50,000+; 8 = $75,000+; 9 = $100,000+; and 10 = $200,000+.

##### Neighborhood Income.

Using zip code data, ABCD has collected median family income in the zip code. We used this variable after dividing it by 5000 to have more understandable beta coefficients.

##### Intracranial Volume.

Using sMRI data, we used total intracranial volume as a covariate.

##### Pubertal Development.

The assessment of pubertal development utilized the Tanner staging method to evaluate the puberty progression in both male and female participants separately. A binary variable was constructed based on parental reports to indicate the pubertal status of the adolescents. The presence of any pubertal development was marked as one, while the absence (pre-pubertal stage) was marked as zero.

### Statistical Analysis

The data analysis was carried out using the SPSS software. Descriptive statistics, including mean and standard deviation (SD), were employed to summarize continuous variables, whereas frequencies were utilized for categorical variables. Pearson’s correlation coefficient was calculated to explore bivariate relationships among all the variables in the study. For the purpose of multivariable analysis, multiple Cox regression analyses were performed with the initiation of tobacco or marijuana use as the dependent variable and the volume of the right nucleus accumbens (NAc) as the independent variable. The analysis confirmed the absence of multicollinearity among the variables. The initial volume of the NAc served as the predictor, with demographic and socio-economic indicators as control variables, and the use of tobacco or marijuana as the dependent outcomes. The analysis was conducted on the combined sample. Due to the high correlation observed between the volumes of the right and left NAc, models were estimated separately for each, rather than combining them into a single model. All the statistical models reached significance at the 0.001 level, with the unstandardized coefficient (b), standard error (SE), 95% confidence interval (CI), and p-value presented for each model. A p-value of 0.05 or lower was considered statistically significant.

### Ethical Considerations

The Adolescent Brain Cognitive Development (ABCD) study’s protocol received approval from the Institutional Review Board (IRB) at several institutions, notably including the University of California, San Diego (UCSD). Consent for participation was obtained from all young participants in the form of assent, along with informed consent from their parents. Our investigation, focusing solely on a secondary analysis of anonymized data, was categorized as exempt from human subjects research, thereby waiving the need for a comprehensive IRB review.

## Results

### Descriptives

A total of 11,795 pre-adolescents, aged 9–10 years, were analyzed. The participants included 6,142 males and 5,653 females. Table 1 presents a summary of categorical descriptive statistics for the adolescents overall. Over the follow-up period, 190 female youths used tobacco, and 91 female youths used marijuana. During the same follow=up period, 234 male youths used tobacco, and 133 male youths used marijuana.

As shown in Table 2, male and female youths did not vary in age, parental education, household income, median zip code income, or right and left NAc volumes. Intracranial volume was larger for males than females.

### Bivariate correlations

Table 3 displays the bivariate, unadjusted correlations, both overall and then separately for females and males. Overall, there was no correlation between right and left NAc volumes and tobacco or marijuana use across the entire sample. Puberty was linked to smaller right and left NAc volumes and was also associated with SES indicators and increased tobacco and marijuana usage. A higher number of SLEs was linked to lower SES, and greater exposure to SLEs was associated with reduced right and left NAc volumes. Additionally, the number of SLEs showed a positive association with tobacco and marijuana usage.

Specifically for females, SLEs were only associated with tobacco use, showing no correlation with right and left NAc volumes or marijuana use. Conversely, for males, SLEs were correlated with both tobacco and marijuana use, as well as with variations in right and left NAc volumes.

The correlation between right and left NAc volumes was consistent for both males and females. However, the correlation between tobacco and marijuana use was stronger among females than males. For females, tobacco use was negatively correlated with right and left NAc volumes. In contrast, for males, marijuana use was positively associated with left NAc volume, but such an association was not observed for females.

### Multivariable Associations (Outcome; NAc Volume)

Table 4 presents the outcomes of linear regression models, where the number of stressful life events (SLEs) serves as the predictor and the volumes of the right and left nucleus accumbens (NAc) are the outcomes, analyzed separately for male and female participants. The models reveal that a higher number of SLEs is associated with a decrease in the left NAc volume in males, but this relationship was not observed in females. Additionally, the number of SLEs did not significantly predict the size of the right NAc volume for either males or females.

### Multivariable Associations (Outcome; Tobacco Use)

As illustrated in Table 5, the volumes of the right and left nucleus accumbens (NAc) were predictive of future tobacco use in both males and females, though the direction of the association differed between sexes. For females, both right and left NAc volumes were negatively associated with the hazard of tobacco use, indicating that larger NAc volumes were linked to a lower risk of initiating tobacco use. For males, on the other hand, only the left NAc volume was significantly associated with the hazard of tobacco use, and this association was positive, suggesting that larger left NAc volumes correlated with a higher risk of initiating tobacco use.

### Multivariable Associations (Outcome; Marijuana Use)

As indicated in Table 6, for males, only the left nucleus accumbens (NAc) volume was positively predictive of future marijuana use, suggesting that larger left NAc volumes were associated with an increased likelihood of initiating marijuana use. The right NAc volume did not show a predictive relationship with future marijuana use for males. For females, neither the right nor the left NAc volumes were associated with the hazard of marijuana use, indicating no significant relationship between NAc volumes and the initiation of marijuana use among female participants.

## Discussion

We found that SLE, the NAcc volume, and future substance use to be correlated, however, these associations depend on sex. A higher number of SLEs predicted a smaller left NAc volume for males but not females. The number of SLEs did not predict the size of the right NAc volume for either sex. Right and left NAc volumes were predictive of future tobacco use for both males and females, but the direction of the association differed. In females, right and left NAc volumes were negatively associated with the hazard of tobacco use, while in males, only left NAc volume was positively associated with the hazard of tobacco use. Left NAc volume was also positively predictive of future marijuana use for males, whereas right NAc volume was not predictive. For females, neither right nor left NAc volumes were associated with the hazard of marijuana use. Finally, SLEs were predictive of marijuana use for males but not females, however, SLEs were predictive of tobacco use for both males and females.

Similar to our observation, literature has also shown a positive association between exposure to SLEs and increased risk of substance use^110,111^. Individuals who are exposed to SLEs also tend to show a more rapid transition to substance use initiation^112^. These suggest that substance use may be used as a coping mechanism for facing SLEs in youth^112^. While other stressors such as perceived discrimination^113^, financial strain, neighborhood stress, and family stress are also shown to increase substance use, a well-established body of evidence has shown that role of SLEs on substance use^110,111^. Some evidence also suggests that youth might be able to reduce their perceived stress by using substances^114,115^. The association between SLE and substance use is also confounded by early puberty^116^, which itself operates as a risk factor of substance use^117–120^.

We found an association between the NAc and substance use. The NAc is a major structure of the brain reward system^33–38^. The NAc’s roles in the reward conditioning, pleasure-seeking, reward dependence, incentive salience, and positive reinforcement have been well established^39–44^. Previous research has also shown changes in the NAc as a predictor of reward seeking behaviors and disorders^45–49^. Past research has shown that NAc change occurs in addiction to food^33,50–52,53^ and drugs^54–57^. Research suggests that NAc changes may explain increased cue sensitivity and reward seeking in individuals with addiction^41,50,58–60^. Animal^61^ and human^62–67^ studies have documented NAc changes in over-sensitivity to reward and the existence of addition^53,62,68–75^. The NAc plays a critical role in driving the desire for rewards, and its alterations are frequently observed in cases of addiction and substance use^61^. The presence of cues related to food and drugs triggers a significant dopamine reaction, heightening the urge for these rewards^53^. Changes in dopamine release within the NAc are linked to increased cravings for food and substances^49,65,76–78^. Despite understanding the NAc’s involvement in addiction development, further research is essential due to several limitations: the majority of insights come from animal research, human studies often involve small participant groups, research settings are usually highly controlled environments, and most investigations are cross-sectional^62–67^. There’s a particular need for extensive national studies that examine the baseline volume of the NAc as an indicator for the onset of substance use among young individuals across diverse demographics.

The NAc has a role in substance use because it is an element of dopaminergic activity in the brain^79^, and is directly involved in seeking and preference of food^80,81–87^ and drugs^121^. NAc regulates motivated behaviors and intake and seeking reward, and response to cues related to food and drugs^81–85^. The Adolescent Brain Cognitive Development study (ABCD)^89^ and other studies^81–85^ show that data suggests that NAc changes predict disorders related to the reward^88–94^.

Our results may have implications for reduction of intergenerational transmission of trauma. A better understanding of the effect of SLE on sex and stress hormones, scholars and therapist may suggest programs and interventions that are tailored to the specific needs of males and females who are exposed to SLEs. Such interventions may aim to reduce the risk of mental distress and substance use following SLE exposure in a sex specific manner. As SLEs reflect structural and societal factors, given their unequal distribution across place, class, SEP, and population groups, our results may suggest that prevention of SLE may have differential return in terms of reducing associated social costs such as substance use of male and female individuals.

Substance use may deliver more reward to youth than adults^120^, a difference that might be due to hyperactivity of regions such as nucleus accumbens combined with reduced inhibition by prefrontal cortex^122,123^. Adolescents show a peak in Nucleus accumbens activity at age 17^124^. Testosterone levels and puberty may increase the reward seeking activity of adolescents^124^, while estradiol levels may decrease functional connectivity between the PFC and the NAc during seeking and expectation of reward^125^.

Our findings are in line with the work by Becker and colleagues. Becker^126^ has written extensively on sex differences in the NAc’s dopamine response. Multiple animal studies have shown that female rats that have undergone ovariectomy display a diminished initial dopamine surge following drug use compared to their castrated male controls. However, when ovariectomized female rats are subjected to estradiol treatment, there is an augmentation of stimulated dopamine release in the dorsolateral striatum, though not in the NAc, resulting in a stark sex-specific imbalance between these mentioned dopaminergic pathways. In instances where drug-taking behavior becomes a habit, dopamine release is reported to be heightened in the dorsolateral striatum and diminished in the NAc. Some of these sex differences may have implications for differences and disparities in addiction between males and females^126^. As such, it is essential to investigate sex differences in the significance of the NAc characteristics in the prediction of substance use in adolescents.

In a study, there was a weaker effect of the number of SLEs on substance use of males than females^127^. In a national study, substance use was more common in females than males among those who were exposed to 3+ SLEs^128^. These studies suggest that while SLE is known to increase the risk of substance use, this effect may not be similar for male and female adolescents.

Programs that can screen youth who are exposed to SLEs, and policies that can reduce exposure to SLEs at school, family, and neighborhood, may be able to reduce youth substance use. Programs that effectively reduce undesired effects of SLEs at schools such as the Cognitive Behavioral Intervention for Trauma in Schools^129^ or the Support for Students Exposed to Trauma^130^ may be of use at the large scale.

Future research on the effects of SLEs on substance use should include mental distress as well as other brain regions and networks. As a unique and rich data set, ABCD is a great opportunity to investigate these processes in the context of youth during adolescence, which represents one of the most vulnerable periods of life. ABCD is a database that has provided an unprecedented opportunity for longitudinal study of developing adolescents in context.

The established impact of SLEs on disrupting normal developmental trajectories underscores the necessity for nuanced comparisons. Specifically, delineating the effects of acute versus chronic and mild versus severe stressors is critical. Moreover, evaluating the influences of various stressor types is imperative. It is equally important to examine the potential moderating roles of family and peer support, resilient personality traits, and other protective factors in buffering against these impacts. Additionally, investigating differences based on gender and sex is essential for a comprehensive understanding of these dynamics.

Our paper also presents an intriguing observation regarding the nucleus accumbens (NAc): a larger volume of the NAc correlates with a decreased risk of initiating tobacco use, whereas an increased NAc volume is associated with higher tobacco use risks among males. This apparent contradiction warrants further exploration to determine whether it represents a spurious association or a biologically grounded effect. Future studies should scrutinize the distinct roles of the right and left NAc within the reward system, particularly in relation to sex differences. Investigating how the brain mediates the impact of SLEs and the protective influence of coping strategies and resilience—both in preventing maladaptive responses and in facilitating recovery through therapeutic interventions—promises to yield significant insights.

## Conclusion

In summary, our findings highlight the links between SLEs, NAc volume, and subsequent substance use, however, these associations are influenced by a complex interplay of sex, brain hemisphere, and substance type.

## Figures and Tables

**Table 1:** Descriptive data of all categorical study variables

	Female (5653)		Male (6142)	
	n	%	n	%
**Puberty**				
No	947	16.8	1617	26.3
Yes	3166	56	3663	59.6
Missing	1540	27.2	862	14
**Parents in the Household**				
One	1517	26.8	1551	25.3
Two	4100	72.5	4535	73.8
Missing	36	0.6	56	0.9
**Tobacco Use**				
No	5436	96.2	5891	95.9
Yes	190	3.4	234	3.8
Missing	27	0.5	17	0.3
**Marijuana Use**				
No	5442	96.3	5931	96.6
Yes	91	1.6	133	2.2
Missing	120	2.1	78	1.3

**Note.** Source: Adolescent Brain Cognitive Development study (ABCD); Sample includes 11,795 pre-adolescents who were followed from age 9–10 for up to 36 months.

**Table 2:** Descriptive data of all continuous

		Female			Male	
	N	Mean	SD	N	Mean	SD
Age (Year)	5648	9.47	0.51	6138	9.49	0.51
Parental education (Jager)	5648	41.75	2.47	6131	41.77	2.45
Total Family Income	5185	7.21	2.43	5604	7.25	2.41
Zip Code Median Income / 50000	5653	0.70	0.46	6142	0.71	0.45
Right NAc Vol	5019	−0.01	0.15	5271	−0.01	0.17
Left NAc Vol	5026	−0.06	0.16	5278	−0.05	0.18
Intra-Cranial Volume (mm^3)*	5462	1444377.24	127439.16	5995	1572453.81	140860.05
SLEs	5460	.5212	1.00544	5937	.5136	1.09051

**Note.** Source: Adolescent Brain Cognitive Development study (ABCD); Sample includes 11,795 pre-adolescents who were followed from age 9–10 for up to 36 months. Nucleus Accumbens: NAc; Stressful Life Events (SLE)

* p < 0.05

**Table 3:** Correlation between study variables overall and by sex

	1	2	3	4	5	6	7	8	9	10	11	12
**All**												
1 Right NAc Volume	1	.635**	−0.014	−0.005	−.022*	.083**	.105**	.070**	.121**	.064**	−.044**	−0.016
2 Left NAc Volume		1	0.008	0.011	−.026**	.075**	.083**	.068**	.105**	.054**	−.053**	−0.001
3 Tobacco Use			1	.276**	.044**	−.059**	−.058**	−.040**	−.058**	−.019*	.040**	.057**
4 Marijuana Use				1	.026**	−.048**	−.044**	−.040**	−.059**	−.039**	.021*	.048**
5 SLEs					1	−.125**	−.051**	−.117**	−.120**	−.062**	.035**	−0.001
6 Married Household						1	.349**	.869**	.551**	.296**	−.068**	0.017
7 Parental Education							1	.270**	.622**	.385**	−.055**	.019*
8 Number of Parents in the Household								1	.511**	.249**	−.049**	0.011
9 Household Income									1	.460**	−.068**	.041**
10 Zip Code Income										1	−.060**	.033**
11 Puberty											1	.111**
12 Age												1
Female												
1 Right NAc Volume	1	.612**	−.039**	−0.014	−0.013	.094**	.123**	.074**	.146**	.070**	−.043**	−0.024
2 Left NAc Volume		1	−.032*	−0.018	−0.014	.086**	.097**	.074**	.126**	.059**	−.039*	−0.005
3 Tobacco Use			1	.311**	.051**	−.061**	−.054**	−.034*	−.047**	−0.012	.040**	.070**
4 Marijuana Use				1	0.014	−.079**	−.053**	−.059**	−.068**	−.052**	.035*	.037**
5 SLEs					1	−.121**	−.055**	−.114**	−.120**	−.036**	.040*	−0.016
6 Married Household						1	.350**	.870**	.549**	.279**	−.076**	0.014
7 Parental Education							1	.265**	.624**	.398**	−.057**	0.015
8 Number of Parents in the Household								1	.505**	.236**	−.060**	0.018
9 Household Income									1	.458**	−.079**	.045**
10 Zip Code Income										1	−.058**	.043**
11 Puberty											1	.175**
12 Age												1
**Male**												
1 Right NAc Volume	1	.620**	0.003	−0.005	−.030*	.073**	.093**	.063**	.104**	.054**	−0.015	−0.019
2 Left NAc Volume		1	.040**	.026*	−.035**	.062**	.073**	.060**	.089**	.044**	−.037**	−0.007
3 Tobacco Use			1	.251**	.038**	−.058**	−.061**	−.045**	−.068**	−.025*	.041**	.046**
4 Marijuana Use				1	.035**	−0.024	−.038**	−.025*	−.053**	−.029*	0.015	.057**
5 SLEs					1	−.130**	−.048**	−.119**	−.119**	−.084**	.031*	0.012
6 Married Household						1	.348**	.868**	.552**	.311**	−.062**	0.020
7 Parental Education							1	.273**	.620**	.373**	−.054**	0.023
8 Number of Parents in the Household								1	.516**	.261**	−.038**	0.004
9 Household Income									1	.461**	−.059**	.036**
10 Zip Code Income										1	−.059**	0.024
11 Puberty											1	.064**
12 Age												1

**Note.** Source: Adolescent Brain Cognitive Development study (ABCD); Sample includes 11,795 pre-adolescents who were followed from age 9–10 for up to 36 months. Nucleus Accumbens: NAc; Stressful Life Events (SLE); Pearson Correlation was used

* p < 0.05

** p < 0.001

**Table 4:** Summary of Linear Regression with Right and Left NAc Volumes as the Outcomes

	B	Std. Error	Beta	95% CI B		p
**Outcome: Right NAc Volume**						
**Female**						
Constant	293.605	25.296		244.013	343.196	< .001
Age	−10.646	2.378	−.057	−15.308	−5.983	< .001
Zip Code Income	1.263	2.651	.006	−3.933	6.460	.634
Intracranial Volume	.000	.000	.375	.000	.000	< .001
SLEs (n)	−.585	1.182	−.006	−2.902	1.732	.621
**Male**						
Constant	279.550	25.639		229.288	329.811	< .001
Age	−7.702	2.386	−.039	−12.379	−3.025	.001
Zip Code Income	−2.754	2.739	−.012	−8.123	2.615	.315
Intracranial Volume	.000	.000	.389	.000	.000	< .001
SLEs (n)	−1.411	1.109	−.015	−3.585	.763	.203
**Outcome: Left NAc Volume**						
**Female**						
Constant	320.811	31.633		258.796	382.825	< .001
Age	−6.530	2.974	−.030	−12.360	−.699	.028
Zip Code Income	5.199	3.315	.021	−1.299	11.697	.117
Intracranial Volume	.000	.000	.222	.000	.000	< .001
SLEs (n)	−1.057	1.478	−.010	−3.954	1.840	.475
**Male**						
Constant	298.810	30.667		238.692	358.929	< .001
Age	−4.435	2.854	−.020	−10.029	1.159	.120
Zip Code Income	−.780	3.276	−.003	−7.202	5.643	.812
Intracranial Volume	.000	.000	.264	.000	.000	< .001
SLEs (n)	−2.583	1.327	−.025	−5.183	.000	.050

**Note.** Source: Adolescent Brain Cognitive Development study (ABCD); Sample includes 11,795 pre-adolescents who were followed from age 9–10 for up to 36 months. Stressful Life Events (SLE); Cox regression is used.

**Table 5:** Summary of Cox Regressions with Right and Left NAx Volume and SLE as the Predictor and Tobacco as the Outcome

	B	SE	Exp(B)	95% CI	for Exp(B)	Sig.
**Outcome: Tobacco**						
**Female**						
Age	.830	.158	2.294	1.685	3.124	< .001
Zip Code Income	−.263	.160	.768	.562	1.051	.100
Intracranial Volume	.000	.000	1.000	1.000	1.000	.767
Right NAc Volume	−.002	.001	.998	.996	1.000	.012
SLEs (n)	.152	.037	1.164	1.082	1.253	< .001
**Male**						
Age	.510	.135	1.666	1.277	2.172	< .001
Zip Code Income	−.338	.145	.713	.537	.949	.020
Intracranial Volume	.000	.000	1.000	1.000	1.000	.632
Right NAc Volume	.000	.001	1.000	.999	1.002	.537
SLEs (n)	.103	.035	1.109	1.035	1.188	.003
**Outcome: Tobacco**						
**Female**						
Age	.844	.157	2.326	1.709	3.165	< .001
Zip Code Income	−.254	.160	.776	.567	1.061	.112
Intracranial Volume	.000	.000	1.000	1.000	1.000	.431
Left NAc Volume	−.002	.001	.998	.997	1.000	.029
SLEs(n)	.154	.037	1.166	1.084	1.255	< .001
**Male**						
Age	.514	.135	1.672	1.282	2.180	< .001
Zip Code Income	−.330	.145	.719	.540	.955	.023
Intracranial Volume	.000	.000	1.000	1.000	1.000	.278
Left NAc Volume	.002	.001	1.002	1.001	1.003	.001
SLEs (n)	.107	.035	1.113	1.039	1.193	.002

**Note.** Source: Adolescent Brain Cognitive Development study (ABCD); Sample includes 11,795 pre-adolescents who were followed from age 9–10 for up to 36 months. Nucleus Accumbens: NAc; Stressful Life Events (SLE); Cox regression is used.

**Table 6:** Summary of Cox Regressions with Right and Left NAx Volume and SLE as the Predictor and Marijuana Use as the Outcome

	B	SE	Exp(B)	95% CI	for Exp(B)	Sig.
**Outcome: Marijuana**						
**Female**						
Age	.722	.225	2.059	1.326	3.198	.001
Zip Code Income	−.828	.222	.437	.283	.674	< .001
Intracranial Volume	.000	.000	1.000	1.000	1.000	.487
Right NAc Volume	−.001	.001	.999	.997	1.002	.574
SLEs (n)	.093	.081	1.097	.937	1.286	.250
**Male**						
Age	.725	.185	2.064	1.435	2.969	< .001
Zip Code Income	−.491	.191	.612	.421	.891	.010
Intracranial Volume	.000	.000	1.000	1.000	1.000	.699
Right NAc Volume	.000	.001	1.000	.998	1.002	.879
SLEs (n)	.110	.046	1.116	1.020	1.220	.016
**Outcome: Marijuana**						
**Female**						
Age	.720	.224	2.055	1.324	3.189	.001
Zip Code Income	−.824	.221	.439	.284	.677	< .001
Intracranial Volume	.000	.000	1.000	1.000	1.000	.502
Left NAc Volume	−.001	.001	.999	.997	1.001	.219
SLEs (n)	.092	.081	1.097	.935	1.286	.256
**Male**						
Age	.731	.186	2.076	1.443	2.987	< .001
Zip Code Income	−.482	.191	.618	.424	.899	.012
Intracranial Volume	.000	.000	1.000	1.000	1.000	.884
Left NAc Volume	.002	.001	1.002	1.000	1.003	.046
SLEs (n)	.113	.046	1.120	1.024	1.224	.013

**Note.** Source: Adolescent Brain Cognitive Development study (ABCD); Sample includes 11,795 pre-adolescents who were followed from age 9–10 for up to 36 months. Nucleus Accumbens: NAc; Stressful Life Events (SLE); Cox regression is used.
